# Nano-Structural Investigation on Cellulose Highly Dissolved in Ionic Liquid: A Small Angle X-ray Scattering Study

**DOI:** 10.3390/molecules22010178

**Published:** 2017-01-21

**Authors:** Takatsugu Endo, Shota Hosomi, Shunsuke Fujii, Kazuaki Ninomiya, Kenji Takahashi

**Affiliations:** 1 Faculty of Natural System, Institute of Science and Engineering, Kanazawa University, Kakuma-machi, Kanazawa 920-1192, Japan; hsmst14@gmail.com (S.H.); fujii-sh091@stu.kanazawa-u.ac.jp (S.F.); 2 Institute for Frontier Science Initiative, Kanazawa University, Kakuma-machi, Kanazawa 920-1192, Japan; ninomiya@se.kanazawa-u.ac.jp

**Keywords:** ionic liquids, cellulose, small angle X-ray scattering, dissolution, composites

## Abstract

We investigated nano-structural changes of cellulose dissolved in 1-ethyl-3-methylimidazolium acetate—an ionic liquid (IL)—using a small angle X-ray scattering (SAXS) technique over the entire concentration range (0–100 mol %). Fibril structures of cellulose disappeared at 40 mol % of cellulose, which is a significantly higher concentration than the maximum concentration of dissolution (24–28 mol %) previously determined in this IL. This behavior is explained by the presence of the anion bridging, whereby an anion prefers to interact with multiple OH groups of different cellulose molecules at high concentrations, discovered in our recent work. Furthermore, we observed the emergence of two aggregated nano-structures in the concentration range of 30–80 mol %. The diameter of one structure was 12–20 nm, dependent on concentration, which is ascribed to cellulose chain entanglement. In contrast, the other with 4.1 nm diameter exhibited concentration independence and is reminiscent of a cellulose microfibril, reflecting the occurrence of nanofibrillation. These results contribute to an understanding of the dissolution mechanism of cellulose in ILs. Finally, we unexpectedly proposed a novel cellulose/IL composite: the cellulose/IL mixtures of 30–50 mol % that possess liquid crystallinity are sufficiently hard to be moldable.

## 1. Introduction

Cellulose—the major component of wood—is renewable, and is the most abundant biopolymer on Earth; therefore, its advanced unitization is crucial for a sustainable society. However, the severe limiting factor is that cellulose does not dissolve in any conventional solvents. A new class of cellulose solvents—ionic liquids (ILs)—was discovered in 2002 [1]. ILs are defined as salts that are liquid around room temperature, and some of them are able to efficiently dissolve cellulose [2,3]. ILs have some outstanding properties as cellulose solvents compared with those used previously: high designability (any appropriate physicochemical properties can be introduced by modifying the cation/anion structures or altering their combination), single component, practically nonvolatile and nonflammable, and highly thermally/chemically/electrochemically stable. Since these green solvents can be a breakthrough for cellulose science, understanding how cellulose dissolves in ILs is a very important issue. Many studies have been conducted thus far to contribute to this aim [2,3,4,5]. The most crucial finding is that IL anions play a major role in the dissolution by disrupting the inter- and intramolecular hydrogen-bonding network of cellulose. These anions mainly interact with the hydrogen of the OH groups in a 1:1 ratio [6], although it was suggested by molecular dynamics (MD) simulations that some anions participate in multiple interactions with the OH groups [7,8]. The role of the cations seems to be less significant; nevertheless, they unquestionably have some impact on the dissolution. Although the definitive role is still controversial, MD simulations have indicated the presence of weak hydrophobic interactions between the cations and the glucose ring of cellulose [4,5], which explains why aromatic cations tend to exhibit high dissolution ability [2,3]. 

**Figure 1:**
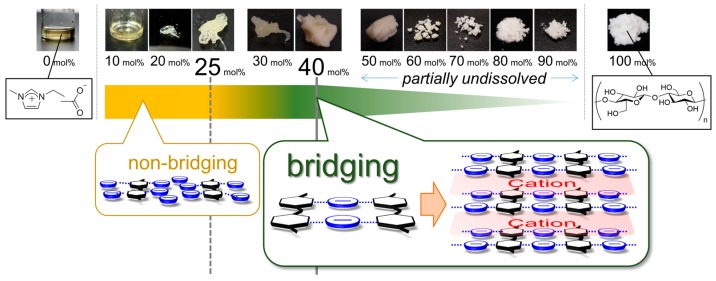
Schematic summary of Reference [9]. Below 25 mol %, the anions mainly interact with the OH groups of cellulose in a 1:1 ratio (non-bridging), while some portion of the anions have multiple interactions with the OH groups [7,8]. In the concentration range of 25–40 mol %, the interaction state of the anion-OH group gradually switches from non-bridging to bridging, which induces the emergence of the cellulose/[Emim][OAc] (1-ethyl-3-methylimidazolium acetate) complex structure. The anions bridge cellulose chains, forming a sheet structure. The sheets are stacked upon each other, intercalated by the cations, forming a layered structure. Note that the exact structure is less ordered than the schematic; i.e., the cellulose chains and the cellulose/anion sheets are not completely straight and flat, respectively. The transformation to the bridging state is completed at 40 mol %, which enables the IL to thoroughly deconstruct the cellulose crystalline structure at this high concentration. The bridging state is maintained above 40 mol %, where undissolved cellulose co-exists. Note that in the concentration range of 10–70 mol %, the mixtures exhibit a lyotropic cholesteric liquid crystallinity, associated with the chain alignment.

It is well known that the concentration of a solution governs the state of dissolution of solute molecules. Particularly at high concentrations, self-assembly phenomena (e.g., lyotropic liquid crystal or micelle formation) are observed. However, a concentration regime of more than 30 mol % (mol % here is defined as glucose unit/(glucose unit + IL))—which is considered to exceed the maximum solubility because the ratio of IL to the OH groups of cellulose is greater than 1:1—has been completely overlooked. Recently, we performed a structural investigation on cellulose dissolved in 1-ethyl-3-methylimidazolium acetate ([Emim][OAc])—as the most representative IL for cellulose dissolution—over the entire concentration range (0–100 mol %) by means of wide-angle X-ray scattering with the aid of quantum chemical calculations and ^13^C solid-state NMR spectroscopy [9]. We demonstrated that the states at concentrations ≥30 mol % showed anomalous deconstruction and restructuring of cellulose, as schematically summarized in Figure 1. Even at 40 mol %, the original crystalline structure of cellulose is absent. Furthermore, a new cellulose/IL complex with high structural periodicity emerges at approximately 35 mol %. It was revealed that these characteristic features originated from the anion bridging phenomenon, which is when one anion interacts with multiple OH groups of different cellulose chains. At concentrations ≤25 mol %, the anion/OH group interaction is mainly 1:1 (non-bridging), as previously reported [6,7,8]. In contrast, the anion bridging state is energetically preferred at the higher concentrations, which induces intriguing structural changes in cellulose.

Although these findings provided a new aspect of cellulose dissolution state in IL, knowledge is limited at the molecular level. It is well known that cellulose exhibits a hierarchical structure [10,11,12]: at the nano-level, it forms microfibrils with the diameter of several nm, which gather together to form larger bundles. From this standpoint, this study is devoted to investigating the structural changes in cellulose/[Emim][OAc] at the nano-level—particularly focusing on the high concentration regime—by means of small angle X-ray scattering (SAXS) measurements, supported by scanning electron microscopy.

## 2. Results and Discussion

**Figure 2:**
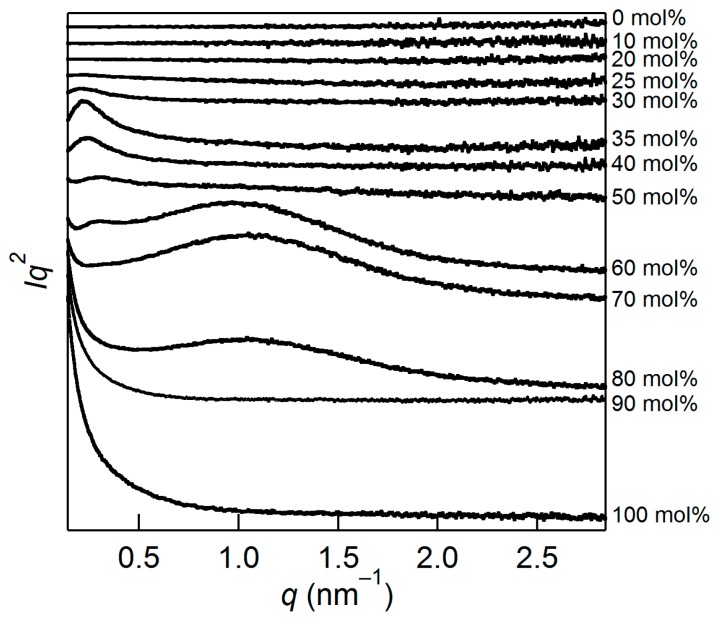
Small angle X-ray scattering (SAXS) patterns of the cellulose/[Emim][OAc] mixtures in the Kratky plot.

**Figure 3:**
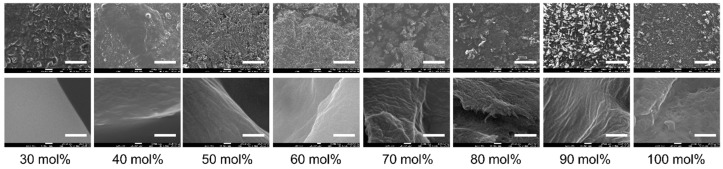
SEM micrographs. White scale bars for the top (100×) and bottom (100,000×) figures represent 300 μm and 300 nm, respectively.

Figure 2 shows SAXS patterns of cellulose/[Emim][OAc] in the Kratky plot (Intensity × *q*^2^ versus *q*) at 0–100 mol % concentration. In this plot, scattering from nanostructures appears as a peak [13]. For pure cellulose (100 mol %), only a shoulder is observable in the plot, indicating the presence of structures larger than the detectable limit of the current SAXS setup (>50 nm). The shoulder scattering would come from bundles of cellulose microfibrils, the presence of which was confirmed by SEM observation (far-right in Figure 3). This shoulder is not observed for the cellulose/[Emim][OAc] mixtures up to a concentration of 40 mol %, even though the upper limit of dissolution at room temperature for this system was reported to be 26–28 mol % [14,15]. This behavior is consistent with the findings obtained at the molecular level [9], and can be explained by the concept of anion bridging. Namely, the anion bridging form—which is energetically preferred around a concentration of 40 mol % (and above)—can completely deconstruct the crystalline structure of cellulose, and consequently, the fiber structures at this concentration. These SAXS results do not contradict the SEM observations shown in Figure 3, while SEM only detects surface morphology. The bundled fibers were observed at 70–100 mol %. At 60 mol %, surface roughness rather than fiber structures was seen. The surface became smoother with decreasing cellulose concentration, and eventually no clear structure was detected at 30 mol %.

There is another characteristic feature in Figure 1, which is the emergence of two new peaks in the concentration range of 30–80 mol %. This clearly indicates the presence of two nanostructures (or aggregations) which are not present in either intact cellulose or [Emim][OAc]. To derive their size and conduct a detailed discussion, the peaks at 1.1 and 0.3 nm^−1^ were fitted with the generalized Guinier–Porod function [16]. The generalized Guinier–Porod function experimentally combines the Guinier and Porod regions, and is expressed as:

(1) $$I\left(q\right)=\frac{I\left(0\right)}{q^{s}}\exp\left(\frac{-q^{2}R_{g}^{2}}{3-s}\right)\;\text{for}\;q\leq q_{1},$$

(2) $$I\left(q\right)=\frac{D}{q^{d}}\;\text{for}\;q\geq q_{1},$$

where

(3) $$q_{1}=\frac{1}{R_{g}}{\left[\frac{\left(d-s\right)\left(3-s\right)}{2}\right]}^{\frac{1}{2}},$$

(4) $$\begin{array}{l} D=I\left(0\right)\exp\left(\frac{-q_{1}^{2}R_{g}^{2}}{3-s}\right)q_{1}^{\left(d-s\right)}\\ =\frac{I\left(0\right)}{R_{g}^{\left(d-s\right)}}\exp{\left[-\frac{\left(d-s\right)}{2}\frac{\left(d-s\right)\left(3-s\right)}{2}\right]}^{\frac{d-s}{2}} \end{array},$$

and where *I*(0) is the scattered intensity at *q* = 0 and *R*_g_ is the radius of gyration. A value of *s* = 0 occurs when the scatterer is a three-dimensional globular object (such as a sphere). For one- (rod) and two-dimensional (lamellae or platelet) objects, the values are defined as 1 and 2, respectively.

**Figure 4:**
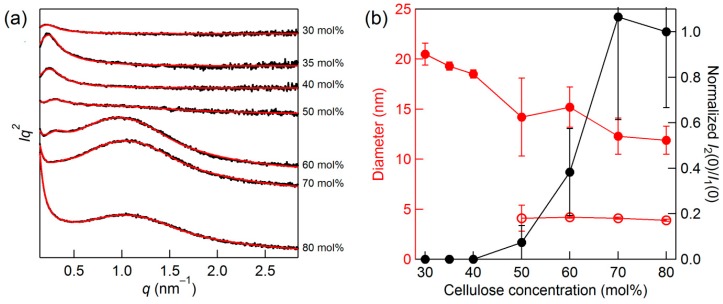
(**a**) SAXS patterns of the cellulose/[Emim][OAc] mixtures in the Kratky plot for the concentrations of 30–80 mol %. Black curves are the observed patterns, while the red curves are the theoretical patterns comprising the sum of two generalized Guinier–Porod functions and one Porod function; (**b**) Red: Diameter changes of the aggregated structures, assuming a sphere. Filled and open symbols are for large and small aggregates, respectively. Black: The normalized *I*_2_(0)/*I*_1_(0) change, where *I*_1_(0) and *I*_2_(0) are the scattered intensities of the aggregates at *q* = 0. The subscripts 1 and 2 are for the large and small aggregates, respectively. The ± signs represent standard deviation produced in the fitting procedures based on a nonlinear regression method.

The fitted results are displayed in Figure 4a. Note that the Porod function was employed for the shoulder scatterer; therefore, the sum of two generalized Guinier–Porod functions and one Porod function was utilized to reproduce the overall patterns. All experimental patterns at the concentrations of 30–80 mol % were well reproduced in the entire *q* range by the combination of the functions. The derived parameters are summarized in Table 1. The *s* values for the aggregates were mostly zero, strongly implying that the shape of these structures was close to a sphere. The diameter of a sphere (*R*) can be determined from *R*_g_ as

(5) $$R^{2}=\frac{5}{3}R_{g}^{2}$$

The concentration dependence of the *R* values is shown in Figure 4b. The behavior of the two structures with varying concentration is quite different. The structure with smaller *R* exists only at 50–80 mol %, and its size is independent of the concentration. In contrast, the structure with larger *R* is observed at 30–80 mol %, exhibiting concentration dependency. The invariant 4.1 nm diameter for the smaller scatterer is reminiscent of the microfibril of cellulose, whose size was reported as 4–5 nm for Avicel [17,18]. It is well known that cellulose fibrillation occurs with mechanical treatment in the presence of solvents [19]. The association of the 4.1-nm-diameter scatterer with the cellulose microfibril is in line with the fact that the shoulder scatterer associated with large bundles also does not exist at concentrations below 50 mol %. If so, then the result demonstrates that the interfibril breakages occur more easily than the intrafibril ones. The dynamic dissolution process can proceed similarly.

**Figure 5:**
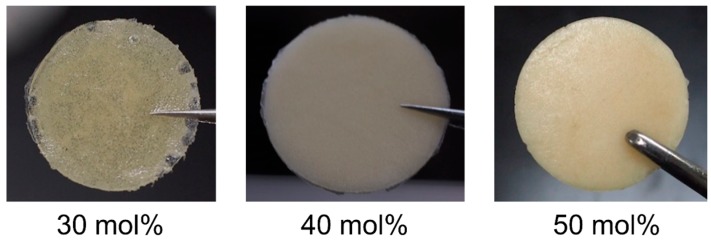
Cellulose/IL (ionic liquid) composite films (30–50 mol %) with a diameter of 25 mm and thickness of 1 mm. The opacity of the film of 40 mol % is attributed to micro air bubbles [9] and surface (or interfacial) roughness, which were confirmed in the 30 mol % film and the SEM images (Figure 3 top). Conversely, the presence of undissolved crystals is also a reason for the opacity at 50 mol %.

The next question is on the origin of the larger scatterer. During the dissolution process, MD simulations observe the peeling of cellulose chains on the fibril surface from the chain edges by intercalation of IL ions [20,21]. Since the intramolecular hydrogen bonding of peeled chains is considerably disrupted by the IL ions, chain bending and twisting occur [22,23]. This flexibility of the terminal parts may enable such chains to entangle, which could correspond to the larger aggregate. This structure disappears at 25 mol %, because all OH groups can interact with an anion in a 1:1 ratio (non-bridging) at this concentration, which unravels the entanglement. Another piece of indirect evidence for the assignment of this structure is the moldability of the cellulose/IL mixture. After several hours of mixing cellulose/[Emim][OAc], the samples of 30–50 mol % intriguingly became mechanically sufficiently hard to be molded (Figure 5), and this moldability disappeared at concentrations below 30 mol %. The idea that cellulose entanglement is the origin of mechanical strength is well-established for cellulose hydrogels [24,25]. Although exploring the potential of this cellulose/IL composite film is beyond the scope of this paper, it is worth noting that among reported cellulose/IL composite materials [26,27,28,29], the emergence of the moldability in cellulose/IL system with liquid crystallinity [9] (in a 10–70 mol % regime) has been discovered here for the first time. The aggregate size decreases with increasing cellulose concentration (Figure 4b), probably because of the decrease in the numbers of glucose units in the chains and the IL ions that participate in the aggregates.

The mixture with 25 mol % cellulose—which gives some scattering but no peak in the Kratky plot—cannot be fitted with the generalized Guinier–Porod model, and thus would need another theoretical model. Rein et al. [15] reported the SAXS profile of the transparent gel-like mixture of cellulose/[Emim][OAc] at 28 mol %, which was prepared by rapid evaporation of a volatile co-solvent. They applied a combination of the Debye–Bueche [30] and Ornstein–Zernike [31] equations for the pattern to gain correlation lengths of the scatterer. The former and latter equations express the scattering from a long-range inhomogeneity and a fluctuation in polymer chains, and were employed to describe the major and minor components in the pattern, respectively. The Debye–Bueche equation is

(6) $$I\left(q\right)=\frac{A}{{\left(1+\xi^{2}q^{2}\right)}^{2}},$$

where *ξ* is the correlation length. The Ornstein–Zernike equation is

(7) $$I\left(q\right)=\frac{A}{1+\xi^{2}q^{2}}.$$

**Figure 6:**
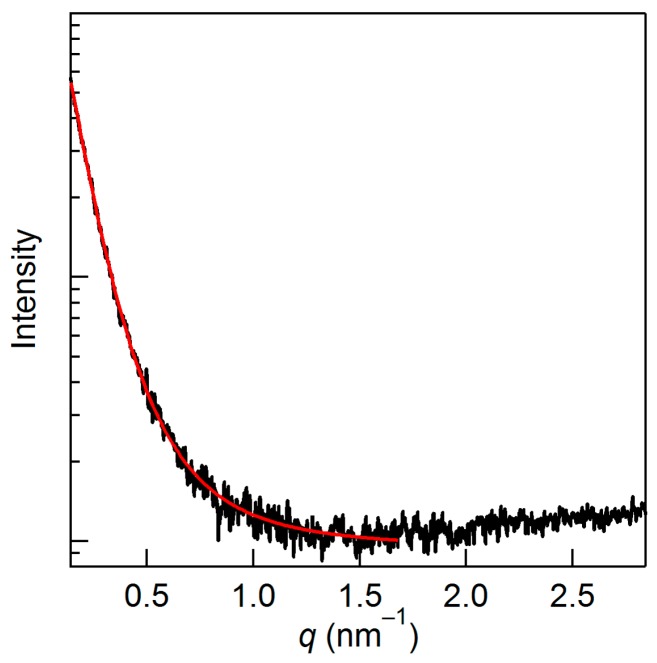
A single logarithmic SAXS pattern of the mixture at 25 mol %. The Debye–Bueche equation was applied (red line) to reproduce the experimental pattern.

The quality of our data for 25 mol % does not require the Ornstein–Zernike equation, and yields a correlation length of 4.8 nm with the Debye–Bueche equation (Figure 6). This value is smaller than the reported value of 18 nm [15], possibly owing to the slightly lower cellulose concentration of our sample and the differences in the sample preparation procedures, whereas the differences in the fitting range of the SAXS pattern and data quality (lower S/N ratio for our results) could contribute to the gap of the correlation length. In any case, it is indicated that the 25 mol % sample does not form definite structures, which were seen in the samples with higher cellulose concentrations.

## 3. Materials and Methods

Microcrystalline cellulose (Avicel PH-101, Sigma-Aldrich, St. Louis, MO, USA) and [Emim][OAc] (>95%, IoLiTec, Heilbronn, Germany) were dried by heating under vacuum (1 Pa) before use. Avicel PH-101 contains approximately 5 wt % of water, and after drying at 343 K for 3 h, the level of water detectable by a moisture analyzer (Sartorius, model MA35) was zero. The water content in the IL was measured using ^1^H-NMR (JEOL JNM-ECS400 or JNM-ECA600, Tokyo, Japan), and no water peak signal was detected after one day of drying at 323 K. The typical sample-mixing procedure was as follows: 0.1 g of Avicel was added to a certain amount of the IL in a vial and mixed with a spatula until the mixture appeared visually homogenous (normally after 5 min). The mixtures were prepared in the range of 10–90 mol % at every 5 or 10 mol %. After one day (which is considered a sufficient period to reach an experimental equilibrium state [9]), the mixture was packed within a Teflon cell with 1 mm thickness. The cell was sandwiched between two sheets of Kapton film (5 μm thickness) and sealed with grease (Apiezon N). These procedures were performed in an inert-atmosphere glove box (the water content was less than 10 ppm). In the mixtures prepared with the current condition, no detectable chemical reactions or cellulose degradation occurred [9].

SAXS measurements were conducted with a NANO-Viewer (IP system, Rigaku Co., Ltd, Tokyo, Japan) with a Cu Kα radiation source (*λ* = 0.154 nm) at an applied voltage of 40 kV and a filament current of 30 mA. The distance between the sample and the detector was set at 700 mm (*q* = 0.142–2.844 nm^−1^). The samples were irradiated for 1 h. The obtained two-dimensional image was transformed into a one-dimensional pattern for theoretical analyses. It should be noted that anisotropic images were not observed in any of our data because the cellulose sample used here was powder. The scattering experiments were conducted three times with a cellulose concentration of more than 40 mol % at different spots in the same sample, and the averaged patterns were used for analyses. This is because there exists inhomogeneity in the sample with such high concentrations due to the presence of undissolved (intact cellulose) regions [9]. Backgrounds including scattering from Kapton films were subtracted from the scattering patterns.

A field emission scanning electron microscope (FE-SEM, JEOL JSM-7100F) was operated at 15 kV. The samples were coated with Au/Pt using an ion sputter coater for SEM observation.

## 4. Conclusions

We have investigated structural changes of cellulose/[Emim][OAc] at the nano-level over the entire concentration range of 0–100 mol % by SAXS measurements with the aid of SEM observations. The shoulder scattering in the Kratky plot ascribed to bundles of cellulose microfibrils does not exist up to 40 mol %. This finding is well explained by the fact that at high concentrations, the anions are energetically preferred to have multiple interactions with OH groups of cellulose chains (anion bridging), which enables the deconstruction of the cellulose crystallinity, and consequently fibril structures at 40 mol %. We observed two aggregated structures that are not observed either in intact cellulose or in the IL at 30–80 mol %. The smaller structure—which is reminiscent of a microfibril with an average diameter of 4.1 nm—does not show concentration dependency. Conversely, the larger one depends on the concentration, and its size is in the range of 12–20 nm. The structure originates from cellulose chain entanglement, which is the origin of the moldability of the cellulose/IL composite films that possess liquid crystallinity.

Since the local concentration of cellulose in ILs at the early stage of the dissolution process should be much higher than that of the consequent solution, the nano-structures observed in the high concentrations can be intermediate states for the dissolution process. Therefore, our findings contribute to an understanding of the dissolution mechanism of cellulose in the IL; inter-fibrillation occurs more easily than intra-fibrillation, and cellulose chains are entangled with each other after IL ions peel the chains from fibrils, which may be one reason for the slow dissolution process of cellulose in ILs.

## Figures and Tables

**Table 1:** SAXS parameters obtained from Figure 4a. The subscripts 1, 2, and *s* are for the large aggregates, small aggregates, and shoulder scattering, respectively. The ± signs represent standard deviation produced in the fitting procedures based on a nonlinear regression method.

Cellulose Concentration/mol %	*R*_g1_/nm	*d*_1_	*s*_1_	*R*_g2_/nm	*d*_2_	*s*_2_	*d*_s_
30	7.9 ± 0.4	4.0 ± 0.0	0.0 ± 0.1	n/a	n/a	n/a	n/a
35	7.5 ± 0.2	3.8 ± 0.0	0.0 ± 0.1	n/a	n/a	n/a	n/a
40	7.1 ± 0.1	3.9 ± 0.0	0.0 ± 0.1	n/a	n/a	n/a	n/a
50	5.5 ± 1.5	3.0 ± 0.3	0.0 ± 0.8	1.6 ± 0.5	3.5 ± 0.4	0.0 ± 0.9	4.0 ± 1.5
60	5.9 ± 0.5	4.0 ± 0.0	0.0 ± 0.0	1.6 ± 0.0	4.0 ± 0.0	0.1 ± 0.1	4.0 ± 0.0
70	4.9 ± 0.2	3.6 ± 0.8	0.1 ± 0.1	1.6 ± 0.0	4.0 ± 0.0	0.1 ± 0.1	4.0 ± 0.0
80	4.7 ± 0.0	3.8 ± 0.4	0.1 ± 0.1	1.5 ± 0.1	4.0 ± 0.0	0.1 ± 0.1	4.0 ± 0.0
